# Maximum standard uptake value of ^18^F-fluorodeoxyglucose positron emission tomography is a prognostic factor for progression-free survival of newly diagnosed patients with diffuse large B cell lymphoma

**DOI:** 10.1007/s00277-012-1602-3

**Published:** 2012-10-19

**Authors:** Yukihiro Miyazaki, Yuichiro Nawa, Masao Miyagawa, Sumiko Kohashi, Koichi Nakase, Masaki Yasukawa, Masamichi Hara

**Affiliations:** 1 Department of Bioregulatory Medicine, Ehime University Graduate School of Medicine, Shitsukawa, Toon, Ehime 791-0295 Japan; 2 Division of Hematology, Ehime Prefectural Central Hospital, Matsuyama, Ehime Japan; 3 Division of Radiology, Ehime Prefectural Central Hospital, Matsuyama, Ehime Japan

**Keywords:** SUV_max_, FDG-PET, Diffuse large B cell lymphoma, Progression, Revised IPI

## Abstract

The treatment of patients with diffuse large B cell lymphoma (DLBCL) would be greatly facilitated with a rapid method for determining prognosis that can be performed more easily and earlier than cytological or specific pathological examinations. It has been suggested that newly diagnosed patients with DLBCL who have low maximum standard uptake value (SUV_max_) on ^18^F-fluorodeoxyglucose positron emission tomography (FDG-PET) are more likely to be successfully treated and remain in remission compared with patients with high SUV_max_, but this concept has been poorly studied. We retrospectively analyzed 50 patients with de novo DLBCL to evaluate the relationship between the SUV_max_ and disease progression. For patients with low SUV_max_ (*n* = 10) and high SUV_max_ (*n* = 40) (*P* = 0.255), respectively, the 3-year overall survival rates were 90 and 72 %, and the progression-free survival (PFS) rates were 90 and 39 % (*P* = 0.012). By multivariate analysis, the revised International Prognostics Index (R-IPI) and SUV_max_ at diagnosis were shown to predict longer PFS. The 3-year PFS for patients with low SUV_max_ classified into the good prognosis group by R-IPI was 100 vs. 62 % for those with high SUV_max_ (*P* = 0.161), and patients with low SUV_max_ classified into the poor prognosis group by R-IPI was 80 vs. 18 % for those with high SUV_max_ (*P* = 0.050). We conclude that the SUV_max_ on FDG-PET for newly diagnosed patients with DLBCL is an important predictor of disease progression, especially for patients with poor prognosis by R-IPI.

## Introduction

Diffuse large B cell lymphoma (DLBCL) is the most common aggressive form of non-Hodgkin’s lymphoma. Combination chemotherapy with rituximab is initially administered to most newly diagnosed patients with DLBCL [1–4]. However, if the patients relapse after the initial chemotherapy, their lymphoma can be poorly managed because most DLBCL cases ultimately become chemotherapy-resistant unless treated with high-dose chemotherapy with autologous stem cell transplantation (ASCT) [5, 6]. Instead of combination chemotherapy with rituximab, other treatments including more intensive chemotherapy, up-front ASCT after the first remission, or other newly developed drugs are required to improve survival of the patients who are at high risk of relapse [7, 8]. Therefore, defining prognostic factors that can easily and accurately classify patients with untreated DLBCL into appropriate risk groups for relapse is highly important for disease management.

Although ^18^F-fluorodeoxyglucose positron emission tomography (FDG-PET) imaging for DLBCL have been widely utilized to evaluate the staging and residual lesions after treatment with high sensitivity [9–12], the relevance of the maximum standard uptake value (SUV_max_) in this technique to disease outcome has been poorly studied. The SUV_max_ at the biopsy site of non-Hodgkin’s lymphoma patients has been reported to correlate with the proliferation potential [13]. Anecdotal cases from our hospital (unpublished) also suggest that patients with low SUV_max_ DLBCL before treatment are more likely to remain in remission, while those with high SUV_max_ DLBCL are more likely to relapse. However, this idea has not been confirmed by a systematic analysis of clinical cases. Therefore, in this study, we retrospectively analyzed 50 patients with DLBCL in order to examine the relationship between the initial SUV_max_ of FDG-PET and disease progression.

## Patients and methods

### Eligibility criteria

In this study, patients with de novo DLBCL, excluding those with transformation from indolent lymphoma, diagnosed between April 2006 and December 2009 at Ehime Prefectural Central Hospital (Matsuyama, Japan) were retrospectively analyzed. FDG-PET imaging was performed in all patients before the treatment, and the SUV_max_ at the primary lesion was measured. The patients subsequently received combination chemotherapy with rituximab. Patients whose primary lesions were excised surgically before FDG-PET imaging or who were treated palliatively, including only radiotherapy or only rituximab, were excluded from this study. The observation period was from April 2006 to March 2011.

### PET/CT acquisition and processing

FDG-PET/CT imaging was performed using a multi-slice PET/CT camera (Discovery STE with 16-slice CT; GE Healthcare). All patients had fasted for a minimum of 6 h, with a blood glucose level of 80–120 mg/dL before intravenous administration of ^18^F-FDG. A whole-body image was obtained exactly 60 min after the intravenous administration of 222–370 MBq (6–10 mCi) of ^18^F-FDG. The PET emission images were corrected for measured attenuation and reconstructed using an ordered-subset expectation maximization iterative algorithm per the manufacturer’s instructions. Integrated PET and CT images were reviewed on Advantage Workstations (GE Healthcare). Display field of view was 60 × 60 cm, which consisted of 192 × 192 matrixes, on the display. Voxel size was 3.125 × 3.125 × 3.27 mm^3^. For each PET data set, in patients with multiple lesions, the tumor with the most intense FDG uptake among all foci was identified by the maximal counts. A volumetric region of interest was approximately 8 cm^3^ (250 voxels) or more and set on the axial fusion images of PET and CT to calculate SUV_max_. A volumetric region of interest encompassing the entire tumor was drawn to ensure correct identification of the maximal counts, and the SUV_max_ was calculated.

### Treatment

All patients received rituximab-containing combination chemotherapy as an initial treatment. The cyclophosphamide, doxorubicin, vincristine, and prednisolone with rituximab (R-CHOP) regimen was administered to younger patients (<70 years old) with DLBCL, and the pirarubicin, cyclophosphamide, vincristine, and prednisolone with rituximab (R-THPCOP) regimen was administered to elderly patients (≥70 years old). Most patients with advanced-stage disease, defined as Ann Arbor stages III or IV, or stage I and II with bulky disease (≥10 cm), received six to eight cycles of R-CHOP or R-THPCOP every 21 days. Only the patients who had Ann Arbor stages I and II without bulky disease received three cycles of R-CHOP or R-THPCOP and field radiation therapy. Complete remission (CR) was defined by FDG-PET scan according to the recently published criteria [9]. All relapsed patients received salvage chemotherapy, and ASCT was performed in eligible cases.

### Statistical analysis

Overall survival (OS) was defined as the time from the start of chemotherapy to death from any cause. Progression-free survival (PFS) was defined as the time from the start of chemotherapy to relapse or death. The Mann–Whitney *U* test was used to calculate the differences between two groups. The probabilities of OS and PFS were estimated by the Kaplan–Meier method. The association of various factors with the hazards of failure for the time-to-endpoint PFS was estimated using the Cox proportional hazard regression model. A *P* value of <0.05 was considered statistically significant. SPSS version 17.0 was used for all analyses.

## Results

### Patients and characteristics

Fifty patients who met the eligibility criteria were analyzed. Clinical characteristics including International Prognostic Index (IPI) factors [14], revised IPI (R-IPI) [15], and the individual treatments are listed in Table 1. Most patients (90 %) received six to eight cycles of R-CHOP or R-THPCOP. Other patients (10 %) received three cycles of R-CHOP or R-THPCOP and field radiation therapy. No patients underwent up-front ASCT after R-CHOP or R-THPCOP. All patients experiencing refractory and relapsed DLBCL (*n* = 26) were treated with salvage chemotherapy, and seven patients (27 %) who achieved complete or partial response and were 70 years old or younger underwent ASCT with high-dose chemotherapy.

**Table 1:** Patients characteristics

	All (*n* = 50)	Low SUV_max_ (*n* = 10)	High SUV_max_ (*n* = 40)	*P* value
Median SUV_max_ (range)	21.0 (8.2–47.1)	11.4 (8.2–14.7)	22.1 (15.0–47.1)	<0.001
Median age (range)	66 (41–85)	63 (49–82)	67 (41–85)	0.280
Male/female	32/18	4/6	28/12	0.080
IPI factors				
Age >60 years	37	8	29	0.632
PS >1	16	1	15	0.099
LDH >normal	32	5	27	0.307
Extranodal site >1	16	4	12	0.548
Stage III/IV	31	6	25	0.885
DLBCL subgroup/subtype				
NOS	42	10	32	
THRLBCL	2	0	2	
PCDLBCL	4	0	4	
PMBL	2	0	2	
IPI H/I, H	30	5	25	0.475
Revised IPI				0.431
Very good	5	2	3	
Good	16	3	13	
Poor	29	5	24	
Bulky mass	5	0	5	0.243
sIL-2R >normal	39	7	32	0.499
Primary lesion				0.401
LNs	24	6	18	
Others	26	4	22	
Treatment				0.161
R-CHOP	36	9	27	
R-THPCOP	14	1	13	
Complete remission	39	9	30	0.311
Recurrence	26	1	25	0.003
Alive/death	38/12	9/1	29/11	0.251

*SUV*_*max*_ maximum standardized uptake value, *IPI* International Prognostic Index, *PS* Eastern Cooperative Oncology Group Performance Status, *LDH* lactate dehydrogenase, *DLBCL* diffuse large B cell lymphoma, *NOS* not otherwise specified, *THRLBCL* T cell/histiocyte-rich large B cell lymphoma, *PCDLBCL* primary cutaneous diffuse large B cell lymphoma, *PMBL* primary mediastinal large B cell lymphoma, *sIL*-*2R* soluble interleukin-2 receptor, *LNs* lymph nodes, *R*-*CHOP* cyclophosphamide, doxorubicin, vincristine, and prednisolone with rituximab, *R*-*THPCOP* pirarubicin, cyclophosphamide, vincristine, and prednisolone with rituximab

The patients were first divided into two prognostic factor groups at various (10, 15, 20, 25, 30, or 35) in order to determine the appropriate cutoff point, and then OS and PFS were analyzed. Although OS curves were not significantly different between each pair of groups for all cutoff values, PFS curves were significantly higher in patients with the SUV_max_ <15 than in those with the SUV_max_ ≥15. Other cutoff values for PFS curves were not statistically significant. Thus, we determined that the SUV_max_ cutoff value should be 15 in this study.

### Univariate and multivariate analysis of OS and PFS for patients with low- and high SUV_max_

The median follow-up time was 32.7 months (range, 4.8–58.3 months). Patients with SUV_max_ <15 (low SUVmax) (*n* = 10) and those with SUV_max_ ≥15 (high SUV_max_) (*n* = 40) had similar backgrounds regarding age, sex, IPI factors, IPI classification, R-IPI classification, and individual treatment (Table 1). The CR rate of all patients was 78 %. CR rates of patients with low SUV_max_ and those with high SUV_max_ were 90 and 75 %, respectively (*P* = 0.311). However, patients with low SUV_max_ had a significantly lower recurrence rate than that of those with high SUV_max_ values (*P* = 0.003).

**Fig. 1:**
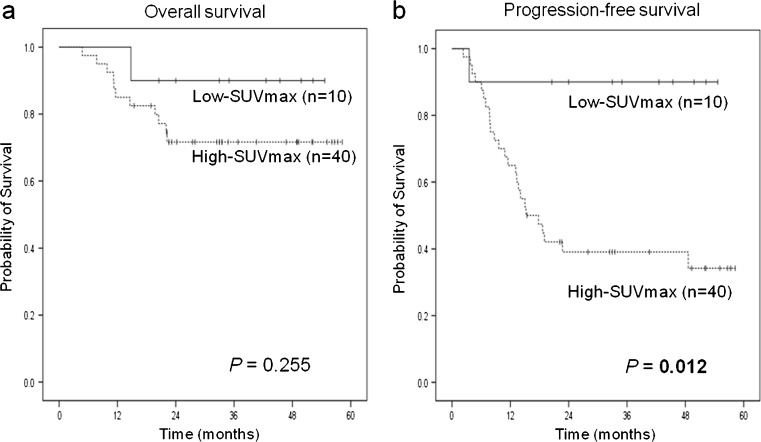
OS (**a**) and PFS (**b**) based on SUV_max_

The 3-year OS rates for patients with low SUV_max_ and for those with high SUV_max_ were 90 and 72 %, respectively (*P* = 0.255) (Fig. 1a). The 3-year PFS rate in each group was 90 and 39 %, respectively (*P* = 0.012) (Fig. 1b). While no factors could predict OS, multivariate analysis of PFS showed that the R-IPI [hazard ratio (HR) 3.37 (1.35–8.39), *P* = 0.009] and low SUV_max_ [HR 7.49 (1.00–55.95), *P* = 0.049] were good independent prognostic factors (Table 2).

**Table 2:** Multivariate analysis of risk factors for OS and PFS

	OS			PFS		
	HR	(95 % CI)	*P*	HR	(95 % CI)	*P*
Revised IPI	1.92	(0.58–6.36)	0.287	3.37	(1.35–8.39)	0.009
Low SUV_max_	2.51	(0.31–20.03)	0.385	7.49	(1.00–55.95)	0.049
Bulky mass	1.02	(0.21–4.99)	0.979	1.29	(0.37–4.51)	0.693
sIL-2R >normal	2.31	(0.27–19.42)	0.441	0.85	(0.27–2.67)	0.775
Primary lesions except LNs	1.49	(0.42–5.23)	0.533	1.01	(0.44–2.29)	0.988

*OS* overall survival, *PFS* progression-free survival, *HR* hazard ratio, *95 % CI* 95 % confidence interval, *IPI* International Prognostic Index, *SUV*_*max*_ maximum standardized uptake value, *sIL*-*2R* soluble interleukin-2 receptor, *LNs* lymph nodes

### Analysis of R-IPI combined with SUV_max_

**Fig. 2:**
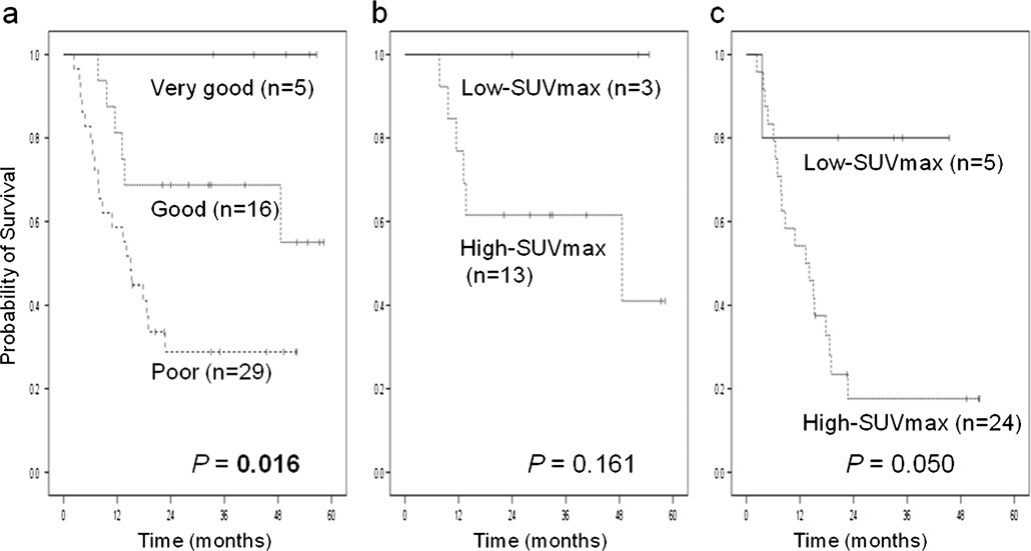
PFS of all patients divided into “very good,” “good,” and “poor” prognosis groups by R-IPI (**a**), of patients in the “good” prognosis group according to SUV_max_ (**b**), and of patients in the “poor” prognosis group according to SUV_max_ (**c**)

According to the R-IPI categories, all patients in this study were divided into prognosis groups of “very good” (*n* = 5), “good” (*n* = 16), and “poor” (*n* = 29), and the 3-year OS and PFS for each group were 100, 81, and 68 % (*P* = 0.167) and 100, 69, and 29 % (*P* = 0.016) (Fig. 2a), respectively. In the very good prognosis group, no patients had a recurrence of DLBCL. In the good prognosis group, the 3-year OS and PFS for patients with low SUV_max_ and those with high SUV_max_ were 100 and 77 % (*P* = 0.386) and 100 and 62 % (*P* = 0.161), respectively (Fig. 2b). In the poor prognosis group, the 3-year OS and PFS for patients with low SUV_max_ and those with high SUV_max_ were 80 and 68 % (*P* = 0.549) and 80 and 18 % (*P* = 0.050), respectively (Fig. 2c).

## Discussion

A relatively higher proportion of patients administered initial chemotherapy including rituximab have been shown to be successfully treated and remain in remission from newly diagnosed DLBCL, compared with those receiving no rituximab [1–4], but a large number of patients still undergo relapses. Treatment strategies other than R-CHOP are either initially more intensive chemotherapy or high-dose chemotherapy followed by up-front ASCT or newly developed drug. More useful prognostic factors are clearly required to identify patients with poorly managed DLBCL. However, most cytological or pathological prognostic factors for patients with DLBCL, such as bcl-2, bcl-6, CD5, CD10, and MUM-1 [16–18], are relatively expensive or time-consuming to implement for general clinical practice, since those measurements can be performed only at specific hospitals or external laboratories.

Our results indicate that the SUV_max_ of FDG-PET in the primary diagnosis of DLBCL is an important predictor of progression after the initial treatment, especially for patients categorized into the poor prognostic group by R-IPI. Furthermore, most patients with low SUV_max_ or categorized into the very good prognostic group by R-IPI sustained CR only with R-CHOP or R-THPCOP therapy. The SUV_max_ has been reported to correlate with the MIB-1 labeling index (i.e., proliferation potential), a known prognostic factor in DLBCL patients treated with R-CHOP [13, 19]. Therefore, DLBCL patients with low SUV_max_ appear to progress more slowly and had better prognosis than those with high SUV_max_. FDG-PET imaging for DLBCL can be used not only for staging but also for predicting the progression after treatment, and the SUV_max_ is highly useful, since it can be measured much more easily and quickly than cytological or specific pathological examinations.

Chihara et al. reported an association between high SUV_max_ on FDG-PET with shorter overall survival in patients with DLBCL [20]. Their conclusions were remarkably similar to ours, while the SUV_max_ cutoff value of 30 in their study differed greatly from that of 15 in our study. The PET/CT camera, incorporation time, and treatment were the same between the two studies. However, more good prognostic patients and fewer poor prognostic patients classified by the IPI score (probably due to patients with SUV_max_ values of 15–30) were included in their report than in our study. Determination of the SUV_max_ cutoff value was likely affected by individual patient characteristics or the number of cases at the different institutes. Therefore, a multicenter analysis may be required in order to define the appropriate SUV_max_ cutoff value.

Collectively, our results suggest that the SUV_max_ on FDG-PET for newly diagnosed patients with DLBCL is an important predictor of PFS. A future prospective study to confirm our results would be of interest.
